# Virulence and Antibiotic Resistance Genes in *Enterococcus* from Wastewater for Reuse and Their Health Impact

**DOI:** 10.3390/microorganisms13051045

**Published:** 2025-04-30

**Authors:** Anthony A. Adegoke, Chibuzor E. Madu, Poovendhree Reddy, Opeyemi K. Fatunla, Thor A. Stenström, Anthony I. Okoh

**Affiliations:** 1 Department of Microbiology, University of Uyo, Uyo P.M.B 1017, Nigeria; 2 Adjunct Researcher, Department of Community Health Studies, Faculty of Health Sciences, Durban University of Technology (DUT), Durban 4001, South Africa; 3 Institute for Water and Wastewater Technology (IWWT), Durban University of Technology (DUT), Durban 4001, South Africa; 4 Department of Community Health Studies, Faculty of Health Sciences, Durban University of Technology (DUT), Durban 4001, South Africa; 5 SAMRC Microbial Water Quality Monitoring Centre, University of Fort Hare, Alice 5700, South Africa

**Keywords:** antibiotic resistance genes, virulence genes, wastewater, *tet* genes, *Enterococcus*, emeA gene, *gelE*

## Abstract

Virulence attributes and putative antibiotic resistance genes from enterococcal isolates from wastewater treatment facilities for sustainable reuse and the areas where they discharge treated water were assessed using phenotypic and molecular methods. This analysis was performed on 269 Enterococci, of which 202 were vancomycin-resistant *Enterococcus* (VRE). VRE strains show markedly higher resistance across multiple antibiotics, especially glycopeptides and beta-lactams, compared to the more susceptible profile observed in vancomycin-susceptible *Enterococcus* (VSE) strains. *vanC* was found in every instance of *E. gallinarum* among VRE and enterococci susceptible to vancomycin (VSE) isolates but not in VR *E. faecium*/*faecalis*. Among VRE, 127 (62.9%) possessed at least one of the *tetK*, *tetL*, *tetM*, or *tetO*, while 22 (17.3%) had two of these genes. The multidrug efflux pump gene *emeA* was detected in 27 out of 202 (13.4%) VRE isolates and 8 out of 67 (11.9%) VSE isolates. Exactly 69 (78.4%) possessed at least one of the virulence determinants tested, with 10 (11.4%) and seven (8%) positive for haemolysis and gelatinase activity respectively. The gelatinase gene, *gelE*, was detected in 16 (18.1%) isolates, while more isolates (*n* = 23; 26.1%) were positive for gelatinase activity. Cytolytic (*cyl*) genes (1.1%), Angiotensin-converting-enzyme genes (*ace*) (13.6%), endocarditis-specific antigen A genes (*efaA*) (25%), hyaluronidase (*hyl*) genes (9.1%), enterococcal surface protein (*esp*) genes (4.5%), among others, were detected. Gelatinase activity and the amplified virulence genes were further validated by sequencing the *gel*-positive amplicons, which were almost identical (98.97%), and the *gelE* gene of *Enterococcus* sp. strain SQ07C was deposited under the GenBank accession number PQ381122. Overall, our results showed that the enterococcal isolates were considered as potential pathogens of notable threat to human health via exposure through reuse, and there is a need for more stringent treatment protocols.

## 1. Introduction

The importance of enterococci as potential pathogens, causing urinary tract infections (UTIs), bacteremia and sepsis, endocarditis, and other infections, includes both their virulence attributes and their resistance to antibiotic therapy [1]. Pathogenic enterococci have long become an emerging public health concern due to their increased resistance to antimicrobial therapy [1,2]. Enterococci, which were ordinarily commensals, became pathogenic due to their ability to acquire genes easily and hence are referred to as emerging pathogens [3] or opportunistic pathogens [4,5]. They are implicated in a variety of infections, and the morbidity and mortality caused by these infections are higher with resistant strains [6,7,8].

Multidrug-resistant enterococci have been detected and reported from wastewater treatment plants [9,10] and from surface waters [10,11]. The antibiotic resistance in enterococci occurs as a result of specific genes conferring resistance. These resistance genes may be intrinsic or acquired, as exemplified in glycopeptide antibiotics such as vancomycin. Some strains of enterococci inherently exhibit a built-in resistance at a fundamental level against beta-lactam antibiotics, such as penicillins, because of their limited binding affinity to penicillin-binding proteins (PBPs) [12]. Alternatively, they may produce the enzyme beta-lactamase and are thus not susceptible to beta-lactam antibiotics like penicillin [13]. Furthermore, it is widely known that *E. gallinarium* is intrinsically resistant to vancomycin. Enterococci can also resist antibiotics by active efflux. Multi-drug efflux pump genes and thirty-four efflux pump genes were reportedly detected in *Enterococcus* [14,15,16]. These genes enhance their resistance to cephalosporins and penicillinase-resistant penicillins, low concentrations of aminoglycosides, clindamycin, fluoroquinolones, trimethoprim-sulfamethoxazole [16]. Enterococci lack cytochrome enzymes, which are necessary for the high-energy production needed for the active uptake of substances such as antibiotics into the cell. This confers some resistance to aminoglycosides at low levels [12]. Even though the genes conferring resistance to ciprofloxacin, *gyrA* and *parC* [17] do exist in enterococci, their resistance to fluoroquinolones has long been shown to be due to active efflux [18].

Between 60 and 80% of enterococci are resistant to tetracycline [10,11]. The resistance is mediated through two different mechanisms related to different genes. The tetracycline resistance genes are *tetL*, *tetM*, *tetN*, and *tetO*, where *tetL* mediates active efflux of tetracycline from cells while *tetM* and *tetN* shield the ribosomes, preventing them from tetracycline [11]. Luna and Robert [19] reported the detection of both *tetM* and *tetO* among *Streptococcus pneumoniae* isolates and predicted the potential for transmission to *Enterococcus faecalis.* Some *tet* genes play essential roles as efflux pump genes, exemplified by *tetA*, *tetC*, *tetE*, *tetG*, *tetK*, *tetL*, etc. Some others exemplified as *tetM*, *tetO*, *tetQ*, *tetS*, *tetT*, *tetW*, etc., are acknowledged as ribosomal protection protein genes, while *tetX* is essentially identified as an enzymatic modification. These genes are detectable in various environmental matrices with attendant public health impact [20,21,22].

Vancomycin has shown activity against a wide range of *Enterococcus* species that are resistant to other antibiotics. The importance of species-specific variations in the carriage of the vancomycin resistance gene among enterococci has long been reported [23]. Further reports [24,25,26] have shown that surveillance of *van* gene carriage among both *Enterococcus* and *Staphylococcus* species is imperative. The expression of these genes leads to the emergence of vancomycin resistance in both genera, which have been categorized with high priority by WHO [27].

Virulence factors that have been previously described in enterococci include gelatinase production, enterococcal surface protein (*esp*), aggregation substance (as), cytolysin, hyaluronidase, and biofilm synthesis [28,29]. While the genes *gelE*, *esp*, *asa1*, *cylA*, and *hyl* are linked to gelatinase, enterococcal surface protein, aggregation substance, cytolysin, and hyaluronidase production, respectively, that of biofilm formation involves several genes that are complex and multifactorial [30]. The biofilms have been associated with multitudes of infections as they afford them the potential to withstand harsh environmental conditions and to circumvent the action of the immune system or antimicrobial agents [31]. Consequently, we assessed the biofilm formation among *Enterococcus* species as well as virulence genes in this study. 

Additional elements linked to virulence in *Enterococcus* encompass endocarditis-specific antigen A (*efa*A), collagen-binding protein (ace), serine protease, capsule, cell wall polysaccharide, and superoxide [3,9]. These virulence factors contribute to infection by facilitating adhesion, colonization, and invasion into host tissues, manipulating host immunity, and producing extracellular enzymes and toxins, thereby amplifying the severity of infections [32]. Adhesins in this context comprise aggregation substance, enterococcal surface protein, and endocarditis-specific antigen A, and collagen-binding proteins help enterococci to adhere to their host’s tissue, while cytolysin, gelatinase, and hyaluronidase affect the host’s tissue, leading to degradation and invasion [32].

The intricate pathways through which virulence and antibiotic resistance genes propagate involve various sources, including agricultural runoff [33], effluents from wastewater treatment plants [34], and improper disposal of pharmaceuticals [35]. These conduits introduce genetic elements into aquatic ecosystems, where they are assimilated by bacteria, endure, and have the potential to transfer to humans and animals. It is imperative to comprehend the migration patterns of virulence and antibiotic resistance genes within recipient environments. Agricultural fields [36], urban zones, and wildlife habitats may act as amplification hotspots for these genes. The interchange of genetic material among environmental bacteria, pathogens, and commensals heightens the risk of disease emergence [37,38]. 

The One Health framework underscores collaborative endeavors across diverse disciplines. Essential to this approach is monitoring and surveillance programs that track the prevalence and movement of genetic elements [39]. Implementing interventions at the source, such as enhanced wastewater treatment [40] and the adoption of sustainable agricultural practices, assumes critical importance in diminishing the influx of virulence and antibiotic-resistance genes into water systems.

Despite notable progress, significant research gaps persist. This research gap includes a paucity of datasets on virulence factors and antibiotic resistance genes borne by *Enterococcus* species recovered from wastewater to be reused in the study area. This gap makes it impossible to fully analyze the potential public health consequences of environmental contamination by such pathogens, particularly in the area under investigation. There is a need for intermittent studies in molecular epidemiology to elucidate the mechanisms of gene transfer, persistence, and their repercussions on public health. Employing such methodologies can augment our comprehension of the diversity and dynamics of virulence and antibiotic resistance genes within water ecosystems. The purpose of this research was to assess the virulence characteristics and antibiotic resistance genes of enterococci extracted from both wastewater treatment plants and the surrounding environment into which they discharge. 

## 2. Materials and Methods

### 2.1. Collecting and Processing of Samples

The gathering of samples from influent, effluent, biofilter/clarifiers, final effluent, and recipient surface water in Durban, South Africa, along with the subsequent isolation and purification process using membrane-Enterococcus Slanetz and Bartley (mSB) agar (Oxoid, UK), was conducted following established bacteriological protocols of Rosenberg-Goldstein and his colleagues as modified, outlined, and cited in Adegoke et al. [10]. The verification of purified isolates involved phenotypical/biochemical characterization, polymerase chain reaction, and Matrix-assisted laser desorption/ionization time-of-flight mass spectrometry. (MALDI-TOF-MS) [10]. The disk diffusion method with modifications as previously outlined [10] was used, while a strain-based antibiogram was depicted using a heatmap plotted with Microsoft Excel 2016.

### 2.2. Detection of Van A, B, C1, and C2/3 Genes Specific to Each Species

Detection of *van* A, B, C1, and C2/3 genes specific to each species was carried out using Multiplex PCR, as established by Iweriebor et al. [41], including PCR primers and conditions, which are described in the Supplementary file (Table S1).

### 2.3. Detection of Other Resistance Genes Tet, gyrA, parC, and emeA Genes

Multiplex PCR was utilized to detect 14 tetracycline resistance (*tet)* genes. The multiplex groups, primer concentrations, and amplification conditions were as adapted by Jia et al. [14]. In Group I, *tet(B)*, the concentration of primers employed was 0.25 µM, while *tet(C)* and *tet(D)* each had primers at a concentration of 0.25 µM and 2.0 µM, respectively. In Group II, *tet(A)*, *tet(E)*, and *tet*(*G*) were each targeted with primers at a concentration of 1.0 µM. Group III included *tet(K)*, *tet(L)*, *tet(M)*, *tet(O)*, and *tet(S)*. 

To identify ciprofloxacin resistance genes, the primer sequences and conditions from Nowroozi et al. [17] were employed for the *gyrA* and *parC* genes. The detection of the *emeA* gene, associated with a multidrug efflux pump, followed the method outlined by Jia et al. [14]. The PCR primers and conditions are described in the Supplementary file (Table S2).

### 2.4. Detection of Virulence Genes

DNA samples obtained from the isolates were assessed for the existence of the *asa1* (Aggregation substance), *gelE* (Gelatinase), *cylA* (Cytolysin), *esp* (Enterococcal surface protein), and *hyl* (Hyaluronidase) using a modified multiplex PCR method based on the approach of Vankerckhoven et al. [29]. This was modified by using extracted DNA from the isolates as the DNA template instead of a whole cell suspension, along with adjustments to the initial denaturation temperature, reaction volume, and gel conditions. Each 25 µL PCR mixture comprised 5 µL of DNA template, 0.1 µM concentration for each primer specific to asa1, *gelE*, and *hyl*, and 0.2 µM concentration for either primer specific to *cylA* and esp. Additionally, 12.5 µL of Hot-Star Taq master mixture (ThermoFisher, Oxford, United Kingdom) was included, consisting of 2.5 U of Hot-StarTaq DNA polymerase, 1.5 mM MgCl_2_, and 200 µM deoxynucleoside triphosphates, with an extra 1.0 mM MgCl_2_. The cycling parameters included an initial activation phase at 95 °C for 5 min, accompanied by 30 cycles of denaturation (94 °C for 60 s), annealing (56 °C for 60 s), and extension (72 °C for 60 s), concluding with a cycle of 10 min at 72 °C.

For the detection of the *ace* and *efa*A genes, monoplex PCR was employed using the primer sequences and cycling conditions from Iweriabor et al. [9]. Each 20 µL of cocktail volume contains 10 µL of master mix, as well as 1 µL of each primer, and 5 µL of DNA template. Cycling conditions for ace included an initial denaturation at 94 °C for 3 min, in which the next was 35 cycles of amplification (93 °C/1 min, 50 °C/1 min, 73 °C/1 min) and a final extension at 72 °C for 10 min. The same conditions were applied for *efa*A, then an annealing temperature of 56.5 °C for 1 min. The resolution of products was conducted as previously described. The primers utilized were described previously by Iweriabor et al. [9] and Vankerckhoven et al. [29].

### 2.5. Validation of Virulence Gene by Sequencing Gelatinase Gene, gelE Amplicon

Successfully amplified products were purified and sequenced using a PCR purification kit and Sanger sequencing. The sequenced data was analysed using BioEdit 7 software (Version 7.7.1) for sequence alignment and to determine the nucleotide sequences, identify genetic variations, compare them with known *gelE* sequences in public databases, and submitted to the National Center for Biotechnology Information (NCBI) Bankit. The phylogenetic reconstruction was performed employing the Neighbor-Joining algorithm, yielding an optimal phylogenetic tree with a cumulative branch length of 2.285. Genetic divergence between sequences was calculated through the Maximum Composite Likelihood approach, with evolutionary distances expressed as the number of nucleotide substitutions per site. Bootstrap support values, indicating the percentage of sites containing unambiguous bases for each clade, are displayed adjacent to internal nodes.

The investigation incorporated 18 protein-coding DNA sequences, analyzing first, second, and third codon positions along with non-coding regions. Ambiguous positions were handled through pairwise deletion during sequence alignment, producing a final dataset containing 1730 homologous sites. All evolutionary computations were executed using MEGA 12 (Version 12.0.11) software.

### 2.6. Statistical Examination

IBM SPSS version 23 was utilized to analyze presumed antibiotic resistance genes through descriptive statistics and correlation analysis. To compare variations across multiple groups, one-way analysis of variance (ANOVA) was utilized, and post-hoc analysis was conducted using the Duncan *t*-test. Statistical value was established with a significance level of *p* ≤ 0.05. Correlation analysis between the tested phenotypic antimicrobials, resistance genes, and virulence genes was performed, and a correlation matrix was plotted using Version 3.8 of Python.

## 3. Results

We analysed 269 enterococci isolates, consisting of 202 Enterococci resistant to vancomycin (VRE) and enterococci susceptible to vancomycin (VSE), which were isolated from sewage and recipient river samples from the two wastewater treatment plants. The 202 VRE consisted of 91 wastewater and 111 river isolates, while the 67 VSE consisted of 36 wastewater and 31 river isolates. 

### 3.1. Antibiotic Resistance Across the Sampling Points and Species

**Figure 1:**
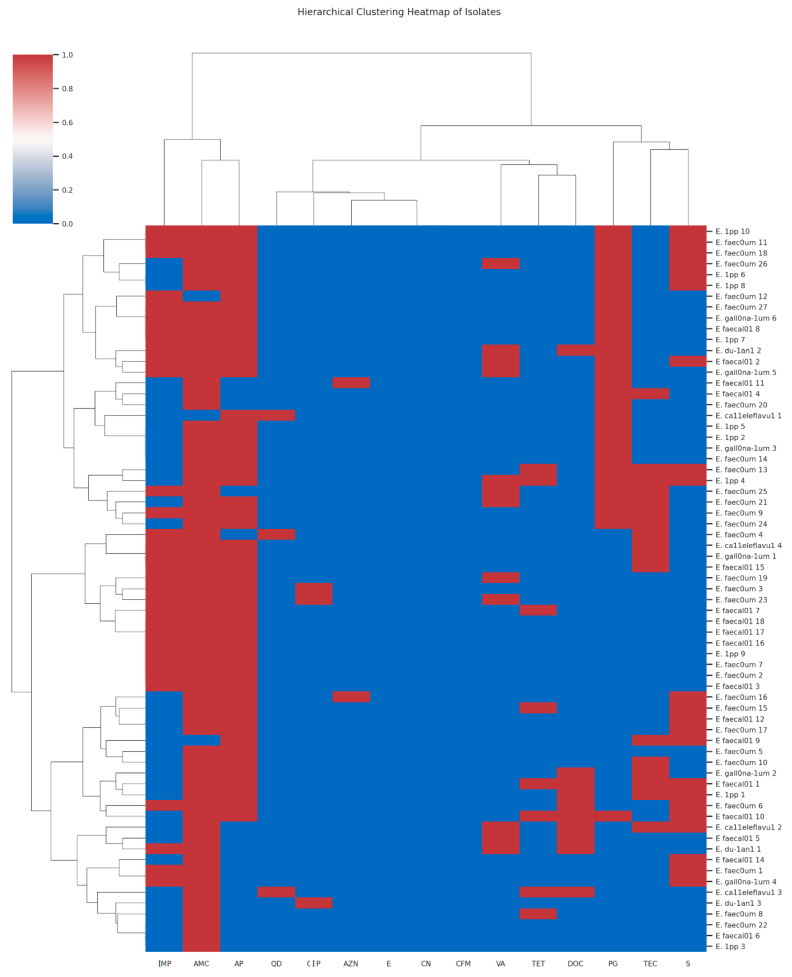
The hierarchical clustering heatmap of the antimicrobial susceptibility patterns of Vancomycin-susceptible *Enterococcus* (VSE) isolates across multiple antibiotics [Red indicates resistance (R), corresponding to a value of −1; white represents intermediate resistance (I), denoted by a value of 0; and blue signifies sensitivity (S), corresponding to a value of 1; The antibiotics CN = Gentamycin; AZN = Aztreonam; CIP = Ciprofloxacin; TEC = Teicoplanin; AMC = Amoxicillin-Clavulanic Acid; VA = Vancomycin; TET = Tetracycline; CFM = Cefixime; S = Streptomycin; E = Erythromycin; QD = Quinupristin-Dalfopristin; IMP = Imipenem; DOC = Doxycycline; AP = Ampicillin and PG = Penicillin G].

**Figure 2:**
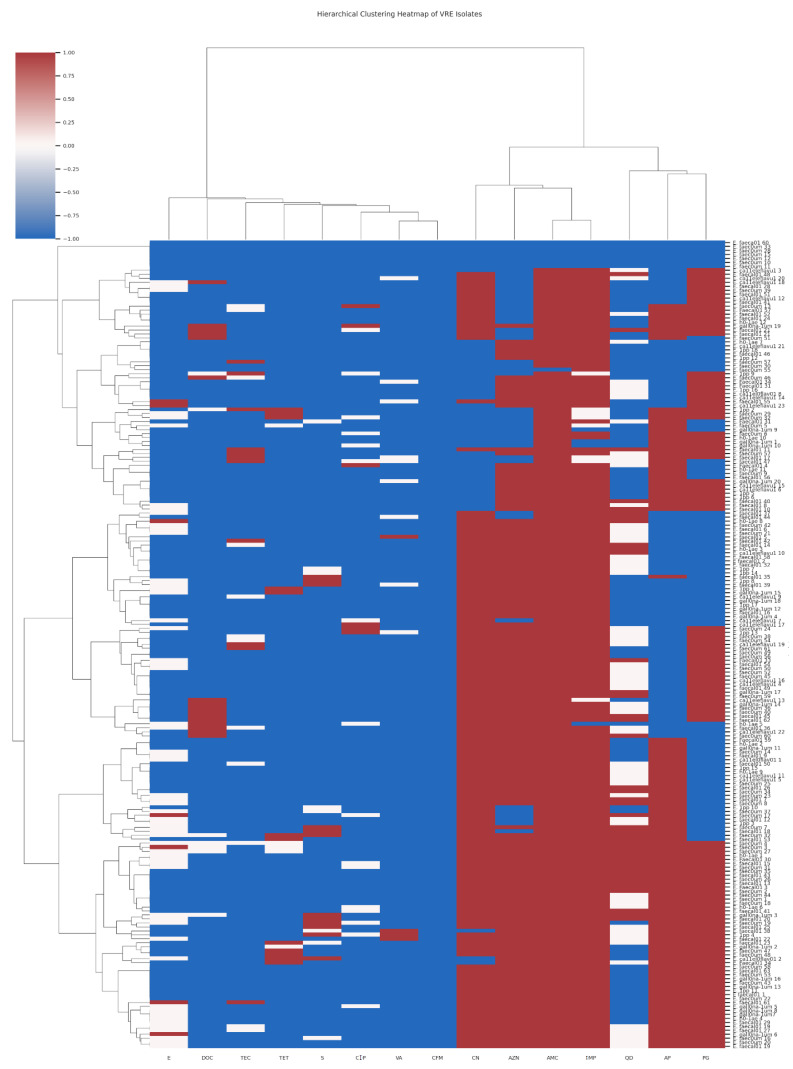
The hierarchical clustering heatmap of the antimicrobial susceptibility patterns of Vancomycin Resistant Enterococcus (VRE) isolates across multiple antibiotics [Red indicates resistance (R), corresponding to a value of −1; white represents intermediate resistance (I), denoted by a value of 0; and blue signifies sensitivity (S), corresponding to a value of 1; The antibiotics CN = Gentamycin; AZN = Aztreonam; CIP = Ciprofloxacin; TEC = Teicoplanin; AMC = Amoxicillin-Clavulanic Acid; VA = Vancomycin; TET = Tetracycline; CFM = Cefixime; S = Streptomycin; E = Erythromycin; QD = Quinupristin-Dalfopristin; IMP = Imipenem; DOC = Doxycycline; AP = Ampicillin and PG = Penicillin G].

The antimicrobial susceptibility testing used for fifteen antibiotics has been previously described [10]. There was no remarkable difference in the resistance pattern of the isolates, which was related neither to the sampling points nor to the species. However, eight isolates exhibited resistance to all fifteen antibiotics assessed. The hierarchical clustering heatmaps (Figure 1 and Figure 2) illustrate the antibiotic susceptibility profiles of *Enterococcus* species, differentiating between Vancomycin-sensitive (VSE) and Vancomycin-resistant (VRE) strains, respectively, across a panel of 15 antibiotics. While both VRE and VSE strains exhibit varying levels of resistance, VRE strains show markedly higher resistance across multiple antibiotics, especially glycopeptides and beta-lactams, compared to the more susceptible profile observed in VSE strains. Seven of these isolates were *E. faecium* and one *E. faecalis*. Five of the seven *E. faecium* super-resistant isolates were isolated from the chlorinated effluent and the clarifiers of Plant II on the same sampling date. The *E. faecalis* was isolated from Plant II influent, while the remaining two *E. faecium* isolates came from Plant I influent.

### 3.2. Vancomycin Resistance Genes Based on Species Diversity

Four different vancomycin resistance genes were detected within the group of VREs. All the VRE isolates except one were shown to possess a *van* gene, with a dominance of *vanA* (73.8%) (Table 1). This gene also occurred within the VSE group, but at a lower frequency. *Van C2/3* occurred in both groups at similar frequencies. In the sensitive group (VSE), no resistance genes were found in most of the isolates (67%). Noteworthy that *vanC* (*vanC1* and *vanC2/*3) was found in all *E. gallinarum* that were VRE and VSE isolates, but not in VRE *E. faecium/faecalis*. In addition, all vancomycin-susceptible *E. gallinarum* possessed at least a *van* gene, but *vanA* was not found in any of the *E. gallinarum isolates. vanC* was detected across all species of VSE. Not more than one *van* gene was identified in the isolates. Worth noting is the detection of VSE *E. gallinarum* from vancomycin-supplemented plates and confirmed disk diffusion test, due to their loss or lack of *vanC1*, known to give them intrinsic resistance.

### 3.3. Detection of Other Resistance Genes

Although there is remarkably high resistance to ciprofloxacin among the isolates, the detection of the two ciprofloxacin resistance genes, *gyrA* and *parC*, was very low. The *gyrA* gene was detected only in 14 VRE isolates, while *parC* was detected in five. Contrastingly, tetracycline resistance genes were detected in greater numbers among the isolates. Four out of the 14 *tet* genes tested were detected, which include *tetK*, *tetL*, *tetM*, and *tetO.* One hundred and twenty-seven (62.9%) of the 202 VRE possessed at least one of these *tet* genes, while 22 (which included the eight isolates that exhibited resistance to all the antibiotics) out of 127 had two *tet* genes (*tetL* and *tetM*). Among the 67 VSE, 21 (31.3%) had at least one *tet* gene, while two *tet* genes were detected in 4 out of 21 (three with *tetL* and *tetM* and one with *tetK* and *tetL*). Multidrug efflux pump gene, *emeA*, was also detected in an appreciable number of the isolates. Table 2 summarizes the detection of the resistance genes.

### 3.4. Detection of gelE and Gelatinase Activity

**Figure 3:**
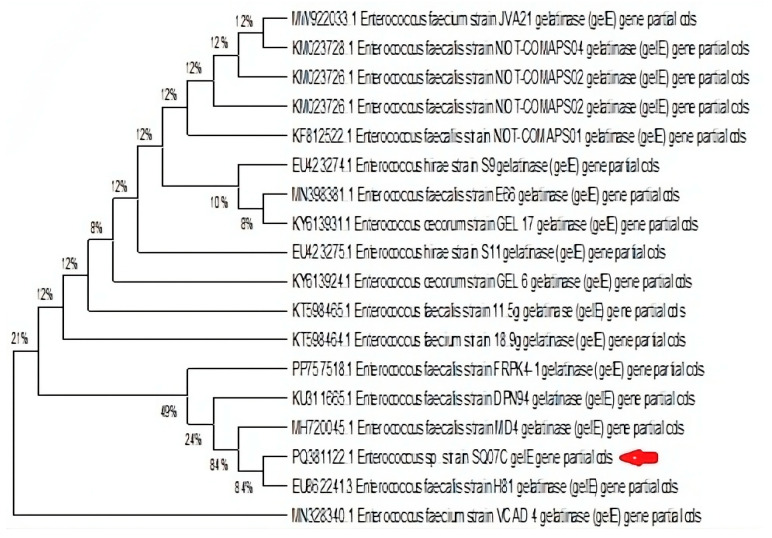
Dendrogram showing hierarchical relationships of *Enterococcus* sp. strain SQ07C (indicated with red arrow) with other referenced strains with gelatinase genes.

Gelatinase gene, *gelE*, was detected in 16 (18.1%) isolates, while 23 (26.1%) were positive for gelatinase activity. Only eight (50%) of the 16 isolates that possessed *gelE* were positive for gelatinase activity, while the additional 15 (65%) isolates that hydrolysed gelatine had no detectable *gelE* gene. The eight isolates that were both positive for *gelE* and gelatinase activity consisted of six *E. faecalis* and two *E. faecium*. However, not all *gelE-positive E. faecalis* were positive for gelatinase activity. The selected sequenced amplicon *Enterococcus* sp. strain Isol_07c_gelatinase_(*gelE*)_gene with accession number PQ381122 validated the presence of this gene as seen in the gel electrophoresis. The dendrogram showing hierarchical relationships of *Enterococcus* sp. strain SQ07C with other referenced strains with gelatinase genes is depicted in Figure 3.

### 3.5. Detection of cylA and Haemolytic Activity

*The cyl* gene was detected only in one isolate (*E. casseliflavus*), which did not show haemolytic activity. Rather, haemolysis was seen in 24 isolates in which the *cyl* gene was not detected. These isolates included *E. faecalis* and *E. faecium* and other species such as *E. gallinarum*, *E. casseliflavus*, and *E. hirae*. 

### 3.6. Detection of Ace, efaA, asa1, hyl, Esp Genes and Biofilm Formation

**Figure 4:**
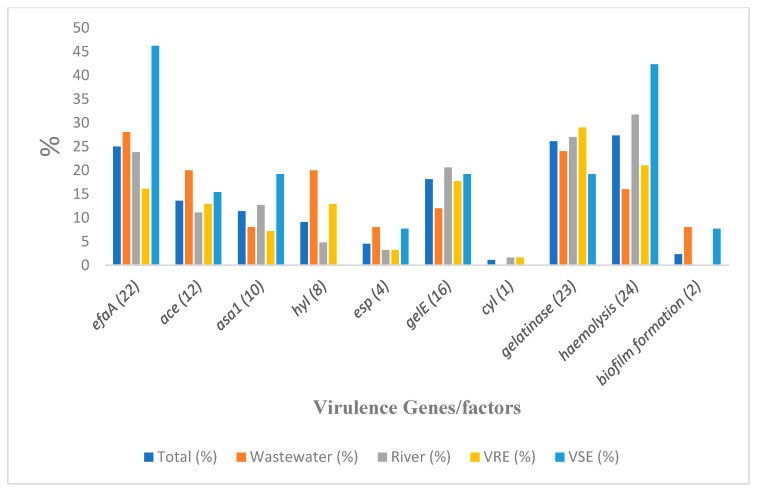
Virulence determinants detected from specific sample sources and Enterococcus isolates’ types. Note: number of isolates from wastewater = 25; river = 63; VRE = 62; VSE = 26.

Angiotensin-converting enzyme genes, *ace*, were detected in 13.6% (12) of the isolates, while endocarditis-specific antigen A genes, *efaA*, were the most prevalent virulence genes among the isolates (25%; *n* = 22). The species that harbored these genes were mostly *E. faecalis*, followed by *E. faecium* and *E. hirae*. The *asa1* was detected in 10 (11.4%) of the isolates, with a majority of *E faecalis*. Hyaluronidase gene, *hyl*, and enterococcal surface protein genes, *esp*, were also detected in 9.1% (8) and 4.4% (4) of the isolates, respectively. The *hyl* was detected only in *E. faecium* isolates, and the *esp* only in *E. faecalis*. The isolates were all either weak or non-biofilm formers except for two isolates, one each of *E. faecalis* and *E. faecium*, which exhibited strong and moderate biofilm formation, respectively. Figure 4 shows the prevalence of virulence genes/factors detected among the isolates, while the virulence genes within different species are presented in Table 3.

Among the 88 isolates, 69 (78.4%) possessed at least one of the virulence determinants tested, with 10 (11.4%) and seven (8%) positive for haemolysis and gelatinase activity respectively, as the only strains with virulence factors and without harboring any of the virulence genes. None of these last two groups included any *E. faecalis* isolates. Furthermore, nine out of the 69 positive isolates were *E. faecalis* and found to be highly virulent, possessing between four and seven virulence genes/factors. There was no notable disparity in the prevalence of any of the six virulence genes (*gelE*, *esp*, *asa1*, *hyl*, *efaA*, and *ace*) between enterococcal isolates when wastewater and river samples were compared (*p* > 0.05), and between VRE and VSE (*p* > 0.05), but the prevalence of these virulence genes among *E. faecalis* and *E. faecium* was significantly different (*p* = 0.05). Figure 4 and Table 3 show the prevalence of the virulence gene among the general isolates and species, respectively. For ease of visualization of tested genes, Figure 4 depicts the virulence determinants detected from specific sample sources (wastewater and river) and their distribution into VRE and VSE isolates. 

**Figure 5:**
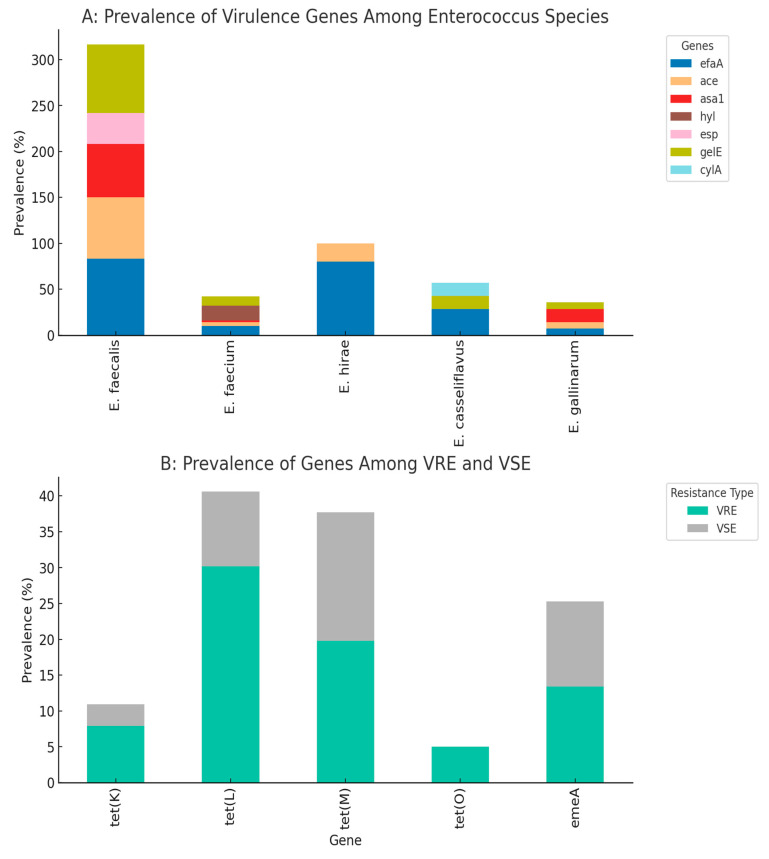
Prevalence of virulence genes among different *Enterococcus* species (Plot (**A**)). Distribution of resistance genes among VRE (Vancomycin-Resistant Enterococci) and VSE (Vancomycin-Susceptible Enterococci) (Plot (**B**)).

**Figure 6:**
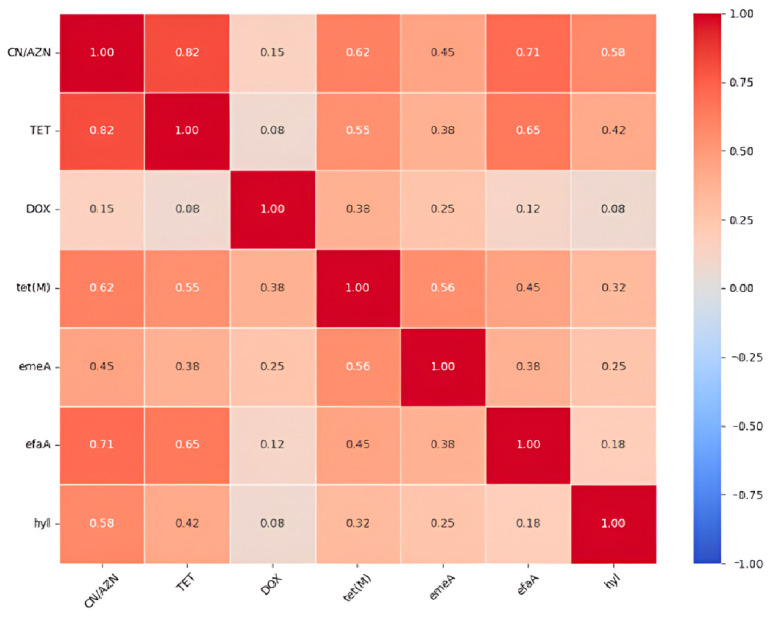
Correlation matrix of the resistance and virulence factors.

Key findings of the correlation analysis revealed co-resistance patterns. There was a strong correlation between gentamycin (CN) and aztreonam (AZN) resistance, which suggests possible cross-resistance or linked resistance mechanisms. The *tet(M)* gene shows the strongest association with phenotypic tetracycline resistance among the tet genes analyzed. In species-specific virulence, *E. faecalis* isolates are strongly associated with *efaA* and *gelE*, while *E. faecium* shows a specific association with the *hyl* gene. Vancomycin resistance associations were also obvious. VRE isolates showed higher prevalence of *tet(L)* (30.2% vs. 10.4% in VSE) and *tet(O)* (5.0% vs. 0% in VSE), suggesting possible genetic linkage or co-selection. Details are depicted in Figure 5, which contains the antibiotic resistance genes and virulence genes. The correlation matrix of the resistance and virulence factors is depicted in Figure 6.

## 4. Discussion

Surface water faces contamination with harmful substances originating from various sources, alongside discharges from wastewater such as runoff from farms, animal waste, and other human-related endeavors [42]. The enterococcal isolates from the river samples may have originated from these sources. The isolates from the two Wastewater treatment plants (WWTPs) and their connected surface water recipients in this study were multidrug resistant. About 50% of all the isolates were resistant to six antibiotics, besides vancomycin and teicoplanin, while 3.4% exhibited outright resistance characteristics to all the antibiotics.

An increasing resistance to antibiotics by enterococci due to the acquisition of resistance genes was noted in publications elsewhere [9,43,44]. Although the intrinsic resistance to quinupristin-dalfopritin by *E. faecalis* [14,45,46] contributed to some extent to the high resistance against this antibiotic, its resistance by *E. faecium* is of concern, as this has been an alternative drug in the treatment of vancomycin-resistant *E. faecium* infections [46]. However, the total resistance against the fifteen antibiotics by eight isolates, consisting of seven *E. faecium* and one *E. faecalis*, is a serious concern [14]. Each of these isolates had *vanA* and two *tet* genes, confirming their increased antibiotic resistance. *E. faecium* and *E. faecalis* are prolific species among others, and their importance and severity in human infection and their antibiotic resistance are well known. *E. faecium* is known for its high resistance to antibiotics, while *E. faecalis* is more virulent [7].

It is known that some strains of enterococci possess an intrinsic resistance to beta-lactam antibiotics such as penicillins due to their low affinity to penicillin binding proteins (PBPs) or by producing the enzyme beta-lactamase against the beta-lactam agents [12,47]. 

The antibiotic resistance patterns observed in *Enterococcus* isolates, particularly *E. faecalis* and *E. faecium*, underscore the importance of a One Health approach to comprehensively address the implications for human and animal health. According to Martin et al. [47], while most *E. faecalis* isolates exhibit susceptibility to penicillin or ampicillin within concentrations of 1 to 8 µg/mL, *E. faecium* isolates demand higher concentrations, typically ranging from 16 to 64 µg/mL for effective growth inhibition. However, certain isolates within these species display heightened resistance, emphasizing the evolving nature of antibiotic resistance.

The study at hand reinforces these findings, revealing resistance rates of 25% to 55% to penicillins among both vancomycin-resistant (VRE) and vancomycin-sensitive (VSE) isolates, primarily dominated by *E. faecalis* and *E. faecium*, with a minor presence in *E. gallinarum*. This highlights the need for collaborative efforts under the One Health paradigm, recognizing the interconnectedness of human and animal health.

The intriguing observation of higher sensitivity to co-amoxiclav, with only 4.5/4.7% resistance in VRE/VSE isolates. Comparing the increased resistance to ampicillin and penicillin G, the findings suggest that clavulanic acid may be effective against penicillinase activity in the same *Enterococcus* isolates. This finding reinforces the importance of a multidisciplinary approach, as it not only sheds light on the mechanisms behind resistance but also suggests avenues for developing interventions that could be beneficial for both human and animal populations [47]. 

Although most of the bacteria exhibited resistance to ciprofloxacin, the frequency of detection of *gyrA* and *parC* genes was low. Thus, the resistance to ciprofloxacin by the enterococci isolates could not be associated with the possession of the corresponding resistance gene. The reason could be either that the genes were present but were not detected or that enterococci resist ciprofloxacin by another mechanism, including active efflux, as shown previously by Mahapatra et al. [18]. This could be partly affirmed by the detection of a number of efflux genes, *emeA* and *tetL*, among the isolates, and has also been shown by other authors [11,14]. Additional mechanisms may also contribute to the high resistance to ciprofloxacin since the number of isolates that possess either *gyrA*, *parC*, or *emeA* genes was lower than those that exhibited resistance to ciprofloxacin.

Enterococci isolates, which did not grow upon sub-culturing on vancomycin-supplemented medium, were considered to be VSE and facilitated the observation of the possession of resistance genetic determinants. In a study by Figueira et al. [48], on characterization of quinolone resistance observed in *Aeromonas* strains originating from aquatic environments, the gene *aacA6-ib-cr* was found in isolates selected with ciprofloxacin. This was similar to the detection of the *vanA* gene in our study. The *van* genes, particularly *vanA*, were detected mostly among VRE, which were isolated on Slanetz and Bartley agar supplemented with vancomycin. However, in the case of the bacteria isolated with antibiotic-supplemented medium, their true resistance profiles to other antibiotics seem to be affected. The antibiotic in the primary isolation medium seemed to play a pre-synergic effect when exposed to other antibiotics during AST, which explains the difference in the resistance pattern to some antibiotics between the VRE and VSE isolates in this study. The isolates from vancomycin-supplemented media were more susceptible to gentamicin, azithromycin, quinupristin-dalfopristin, and imipenem than those from non-vancomycin media. This also could be due to the fact that the organism must have been weakened by the antibiotics in the primary medium and then succumbed more easily to some other antibiotics during AST. Furthermore, the synergic effect of vancomycin with an aminoglycoside [49] and quinopristin-dalfopristin with a cell wall-active agent [45] against enterococci has been shown.

The detection of the *vanA* gene on some VSE isolates may seem unexpected. The presence of these genes in organisms without subsequently being expressed is well documented [50]. However, resistance marked by these isolates upon AST could be due to the fact that the putative *vanA* gene was not actively expressed. This slow expression could lead to misinterpretation. Enterococci isolates, which did not grow upon sub-culturing on vancomycin-supplemented medium, were taken to be VSE. Therefore, those isolates, which carry *van* genes that were poorly expressed, may not grow on vancomycin-supplemented medium and thus be classified as VSE. Hence, upon AST, following the standard reference guideline, the measured zones of inhibition were not large enough to be interpreted as susceptible and thus were regarded as resistant. Therefore, for proper determination of antibiotic resistance/susceptibility of an organism, both AST and detection of resistance genes, when possible, should be employed.

*Enterococcus* species is the third-most etiological agent of hospital-based infections globally [51] due to their virulence attributes coded for by virulence genes.

It is noteworthy to state that the isolates revealed the presence of seven virulence genes, namely *ace*, *asa1*, *cylA*, *efaA*, *esp*, *gelE*, and *hyl*, with 74% of the isolates positive for at least one virulence gene/factor. The *efaA* gene was the most prevalent (25%) while the *cylA* gene was the least (1.1%) virulence gene detected. In as much as the *cylA* gene was the least prevalent, hemolysin (27.3%) was the highest among the three virulence factors detected. In addition, the number of isolates positive for gelatinase activity was higher than the number of isolates in which the associated gene (*gelE*) was detected. Therefore, there may be the possibility of false negative results in the detection of the virulence genes. The detection of the *gelE *gene in enterococcal strains does not correlate with the ability to produce gelatinase, as shown in several investigations [30,50,52]. This was also the situation in this study, where only 50% of the isolates in which the *gelE* gene was detected actually expressed gelatinase. This could be a result of “silent genes”. However, the *E. faecalis fsr* quorum-sensing system was shown to control the production of gelatinase [50]. Disabling the *gelE* gene, which is regulated by fsr and encodes the zinc-metalloprotease known as gelatinase [53], led to *gelE* not being expressed. *gelE* is shown to be associated with *E. faecalis* [29], but Lopes et al. [54] mention that *gelE* was observed in species different from either *E. faecalis* or *E. faecium*, which was also the case in this study.

The cytolysin operon contains five genes: *cylL1*, *cylL2*, *cylM*, *cylB*, which are responsible for the physical effect of the L component, and *cylA* for expressing the activator protein A [29]. This activator (A) is a serine protease, which is responsible for further cleavage and activation of *cyl1* and *cyl2* subunit extracellularly for subsequent full expression [55,56]. In this study, detection of the *cylA* gene was chosen (because of the role of the activator protein A in cytolysin expression) rather than the whole cytolysin gene operon. Cytolysin is associated with haemolytic activity [55,56,57]. Thus, β-haemolytic isolates indicate possession of *cylA.* However, β-haemolysis in 24 isolates and the detection of the *cylA* gene only in one isolate could not be linked. Either the isolates have this gene, but it was not detected in the PCR used, or the haemolytic activity of these isolates is a result of factors other than cytolysin.

Although Comerlato et al. [5] reported that there was no association between the connection of the origin of isolation and the existence as well as the functionality of any virulence factor, some virulence attributes may be more common in enterococci isolated from a particular kind of infection [58]. Certain adhesins, particularly *agg*/*asa*1 and *efa*A, showed notably higher occurrence within non-invasive *Enterococcus* spp. Isolates, such as in the reported genital tract infection (GTI) isolates, wound isolates, and isolates from urinary tract infections (UTIs), in contrast to isolates causing invasive bacteremia [32]. The ace/*acm* exhibited a notably increased prevalence by enhancing the potential to invade their host, as opposed to those from non-invasive genitourinary and wound sources. However, for environmental isolates, the type and frequency of virulence genes might not differ among isolates from different sites. This study did not show any difference in the type and the occurrence of virulence genes among the isolates from wastewater treatment plants and those of the river isolates. Previous reports have shown no differences in the presence of virulence genes in both VRE and VSE, in accordance with this study [5]. However, since Biswas et al. [30] showed a higher prevalence of *hyl* gene among VRE than VSE, the detection of *hyl* genes only among the *E. faecium* isolates of VRE may be considered relevant. These pathogens and their genes may constitute serious human health hazards and the development of superbugs [59]. 

The detection of vancomycin-susceptible *E. gallinarum* is notable, despite the expected intrinsic resistance, and is in line with observations made at different times by other researchers [60,61]. The *vanC1* gene, located on its chromosome, mediates a mechanism of low resistance; its clinical detection and interpretation are challenging [62]. The vanC1 gene leads to the production of a D-Ala-D-Ser peptidoglycan precursor that is altered and of lowered affinity for vancomycin, yielding minimum inhibitory concentrations (MICs) typically 4–32 µg/mL [63]. Despite this natural resistance, existing evidence supports phenotypic heterogeneity with decreased MIC isolates that may be vancomycin susceptible on regular testing and effective therapeutic applications [61]. This necessitates the genotypic confirmation while identifying van genes in vancomycin resistance studies in *E. gallinarum*, as was performed in this study.

Exposure pathways of Enterococcus in wastewater may most likely be occupational [64], though residents of close settlements to the WWTPs may inhale aerosols containing virulence/antibiotic gene harboring *Enterococcus* from the WWTPs in a form of domestic exposure [2,65]. Such aerosols may settle in exposed food or, rarely, possibly on open wounds of the residents [66]. Using their virulence genes like those reported in our studies among others, both *E. faecalis* and *E. faecium* are recognized for 9 out of 10 clinical cases [6,67], because the virulence genes, when expressed, enhance their degree of pathogenicity. On the global level, these two species of Enterococcus are number 3 and 4 in terms of their pathogenicity [68]. The Cytolytic (*cyl*) and endocarditis-specific antigen A genes detected in our study make the bacteria a public health concern for invasion of the blood and organs. For example, *Enterococcus* species have been placed as the third most prevalent aetiological agent of bacteremia (morbidity: approx. 11–13%) in Europe and North America [69,70]. Gelatinase gene, *gelE*, which was detected in some of our isolated *Enterococcus* species, might play an active role in colonizing a wound when such a wound is exposed to contaminated water. *gelE* codes for gelatinase enzymes, which play vital roles in biofilm formation and wound colonization [71]. Enterococci are highly prevalent in wound infections [72,73]. So, there is a high risk associated with the reuse of wastewater effluent containing such bacteria for recreation, where individuals with wounds may be exposed. 

The Cytolytic (*cyl*), Gelatinase (*gelE*), and Endocarditis-Specific Antigen A genes play pivotal roles in bacterial pathogenesis. The *Cyl* gene, associated with hemolysin production, contributes to the virulence of various bacteria, impacting both human and animal health. Gelatinase, encoded by *gelE*, facilitates tissue invasion and immune evasion, influencing the severity of infections. These virulence factors are particularly significant in *Enterococcus* species, where they contribute to the pathogenesis of infectious diseases in humans and animals.

The Endocarditis-Specific Antigen A (*cylA*) gene serves as a marker for endocarditis, a condition affecting both humans and animals, highlighting its relevance in the One Health perspective that emphasizes the interconnectedness of human, animal, and environmental health. Research by Sava et al. [74] demonstrates the importance of these genes in the context of *Enterococcus* infections, showcasing their impact on both human and veterinary medicine. By unraveling the role of *Cyl*, *gelE*, and endocarditis-specific antigen A genes (*cylA*), this knowledge can inform strategies for disease surveillance, prevention, and treatment, aligning with the holistic One Health perspective that emphasizes the interconnectedness of human, animal, and environmental health.

The previously identified and reported *Enterococcus* species were further analysed for virulence genes and antibiotic resistance genes. Various species of Enterococci possess both genes and phenotypic virulence attributes in various patterns. Since organisms possessing virulence genes or expressing virulence factors are potentially virulent. 

## 5. Conclusions

It can be concluded that most of the enterococcal isolates from wastewater and river samples from the study depicted phenotypic and genotypic virulence attributes, and thus, they are considered a potential risk to the health of the community that reuses the water. With the majority of the isolates coming from river samples, the reuse of this water for irrigation or other activities may contribute significantly to the spread of these pathogenic and resistant enterococci. It may also lead to infection through direct or indirect exposure. Therefore, frequent surveillance is essential for appropriate mitigation.

## Figures and Tables

**Table 1:** Vancomycin resistance genes were detected among species of VRE and VSE.

Group	*Van* Gene	No of Isolates	Percentage	Species Involved
VRE	*vanA*	149	73.8	*Enterococcus faecium*, *Enterococcus faecalis*, *Enterococcus hirae*, *Enterococcus durans*
	*vanB*	3	1.5	*E. faecalis*
	*vanC1*	33	16.3	*E. gallinarum*, *E. casseliflavus E. cecorum*
	*vanC2/3*	16	7.9	*E. gallinarum*, *E. casseliflavus E. faecium*
VSE	*vanA*	14	20.9	*E. faecium*, *E. hirae*, *E. faecalis*, *E. durans*
	*vanB*	0	0	no species
	*vanC1*	1	1.5	*E. casseliflavus*
	*vanC2/3*	7	10.4	*E. gallinarum*, *E. casseliflavus E. faecium/faecalis*
	none	45	67.2	*E. faecium*, *E. hirae*, *E. faecalis*, *E. durans*

VRE = enterococci resistant to vancomycin; VSE = enterococci susceptible to vancomycin.

**Table 2:** Tetracycline resistance and efflux pump responsible for multiple drug resistance genes detected in Enterococcal isolates.

	VRE No (%)	VSE No (%)
*tet(K)*	16/202 (7.9)	2/67 (3)
*tet(L)*	61/202 (30.2)	7/67 (10.4)
*tet(M)*	40/202 (19.8)	12/67 (17.9)
*tet(O)*	10/202 (5.0)	0 (0)
*emeA*	27/202(13.4)	8/67 (11.9)

**Table 3:** Prevalence of the virulence genes and the *Enterococcus* species involved.

Virulence Gene	*E. faecalis* (12)	*E. faecium* (50)	*E. hirae* (5)	*E. casseliflavus* (7)	*E. gallinarum* (14)
*efaA*	10 (83.3%)	5 (10%)	4 (80%)	2 (28.6%)	1 (7.1%)
*ace*	8(66.7%)	2(4%)	1 (20%)	-	1 (7.1%)
*asa1*	7 (58.3%)	1 (2%)	-	-	2 (14.3%)
*hyl*	-	8 (16%)	-	-	-
*esp*	4 (33.3%)	-	-	-	-
*gelE*	9 (75%)	5 (10%)	-	1 (14.3%)	1 (7.1%)
*cylA*	-	-	-	1(14.3%)	-

## Data Availability

All data supporting the findings of this study are available within the paper and its Supplementary Information. The list of primers for the detection of resistance genes is provided in Supplementary Table S1, while Primers for the detection of virulence genes are presented in Table S2, along with the original reference used in this study. The data presented in this study are openly available in [GenBank] at NCBI, accession number PQ381122.

## Supplementary Materials

The following supporting information can be downloaded at: https://www.mdpi.com/article/10.3390/microorganisms13051045/s1. Table S1: List of primers for detection of resistance genes; Table S2: Primers for the detection of virulence genes.

## References

[1] Tendulkar S.R., Baghdayan A.S., Shankar N. (2006). Putative surface proteins encoded within a novel transferable locus confer a high-biofilm phenotype to *Enterococcus faecalis*. J. Bacteriol..

[2] Adegoke A.A., Stenstrom T.A., Rose J.B., Jiménez-Cisneros B., Mihelcic J.R., Verbyla M.E. (2019). Septic Systems. Global Water Pathogen Project.

[3] Giridhara Upadhyaya P.M., Ravikumar K.L., Umapathy B.L. (2009). Review of virulence factors of Enterococcus: An emerging nosocomial pathogen. Indian J. Med. Microbiol..

[4] Al-Ahdal M.N., Abozaid S.M., Al-Shammary H.F., Bohol M.F., Al-Thawadi S.I., Al-Jaberi A.A., Senok A.C., Shibl A.M., Al-Qahtani A.A. (2012). Characterization of *Enterococcus faecium* isolates and first report of *van*B phenotype–*van*A genotype incongruence in the Middle East. Eur. J. Clin. Microbiol. Infect. Dis..

[5] Comerlato C.B., de Resende M.C.C., Caierão J., Alves d’Azevedo P. (2013). Presence of virulence factors in *Enterococcus faecalis* and *Enterococcus faecium* susceptible and resistant to vancomycin. Mem. Inst. Oswaldo Cruz.

[6] Billington E.O., Phang S.H., Gregson D.B., Pitout J.D.D., Ross T., Church D.L., Laupland K.B., Parkins M.D. (2014). Incidence, risk factors, and outcomes for *Enterococcus* spp. bloodstream infections: A population-based study. Int. J. Infect. Dis..

[7] Fisher K., Phillips C. (2009). The ecology, epidemiology, and virulence of Enterococcus. Microbiology.

[8] Diazgranados C.A., Zimmer S.M., Klein M., Jernigan J.A. (2005). Comparison of mortality associated with vancomycin-resistant and vancomycin-susceptible enterococcal bloodstream infections: A meta-analysis. Clin. Infect. Dis..

[9] Iweriebor B.C., Obi L.C., Okoh A.I. (2015). Virulence and antimicrobial resistance factors of *Enterococcus* spp. isolated from faecal samples from piggery farms in Eastern Cape, South Africa. BMC Microbiol..

[10] Adegoke A.A., Madu C.E., Reddy P., Stenström T.A., Okoh A.I. (2022). Prevalence of vancomycin-resistant Enterococcus in wastewater treatment plants and their recipients for reuse using PCR and MALDI-ToF MS. Front. Environ. Sci..

[11] Molale L.G., Bezuidenhout C.C. (2016). Antibiotic resistance, efflux pump genes and virulence determinants in *Enterococcus* spp. from surface water systems. Environ. Sci. Pollut. Res. Int..

[12] Klare I., Konstabel C., Badstübner D., Werner G., Witte W. (2003). Occurrence and spread of antibiotic resistances in *Enterococcus faecium*. Int. J. Food Microbiol..

[13] Miller W.R., Munita J.M., Arias C.A. (2014). Mechanisms of antibiotic resistance in enterococci. Expert Rev. Anti-Infect. Ther..

[14] Jia W., Li G., Wang W. (2014). Prevalence and antimicrobial resistance of Enterococcus species: A hospital-based study in China. Int. J. Environ. Res. Public Health.

[15] Davis D.R., McAlpine J.B., Pazole C.J., Talbot M.K., Alder E.A., White C., Jonas B.M., Murray B.E., Weinstock G.M., Rogers B. (2001). *Enterococcus faecalis* multi-drug resistance transporters: Applications for antibiotic discovery. J. Microbiol. Biotechnol..

[16] Jonas B.M., Murray B.E., Weinstock G.M. (2001). Characterization of emeA, a NorA homolog and multidrug resistance efflux pump, in *Enterococcus faecalis*. Antimicrob. Agents Chemother..

[17] Nowroozi J., Akhavan Sepahi A., Sabokbar A. (2014). Comparison of gyrA and parC mutations in ciprofloxacin-resistant and -susceptible *Enterococcus faecalis* isolates. J. Med. Microbiol..

[18] Mahapatra A., Raj Kumar Patro A., Khajuria A., Dhal S., Praharaj A.K. (2022). Ciprofloxacin-resistant Gram-negative isolates from a tertiary care hospital in Eastern India with novel gyrA and parC gene mutations. Med. J. Armed Forces India.

[19] Luna V.A., Roberts M.C. (1998). The presence of the tet(O) gene in both tetracycline-resistant and -susceptible strains of Streptococcus pneumoniae. J. Antimicrob. Chemother..

[20] Koike S., Krapac I.G., Oliver H.D., Yannarell A.C., Chee-Sanford J.C., Aminov R.I., Mackie R.I. (2007). Monitoring and source tracking of tetracycline resistance genes in lagoons and groundwater adjacent to swine production facilities over a 3-year period. Appl. Environ. Microbiol..

[21] Macauley J.J., Qiang Z., Adams C.D., Surampalli R., Mormile M.R. (2007). Disinfection of swine wastewater using chlorine, ultraviolet light and ozone. Water Res..

[22] Stenström T.A., Okoh A.I., Adegoke A.A. (2016). Antibiogram of environmental isolates of Acinetobacter calcoaceticus from Nkonkobe Municipality, South Africa. Fresenius Environ. Bull..

[23] Mahmoudpour A., Rahimi S., Sina M., Soroush M.H., Shahisa S., Asl-Aminabadi N. (2007). Isolation and identification of *Enterococcus faecalis* from necrotic root canals using multiplex PCR. J. Oral Sci..

[24] Mundy L.M., Sahm D.F., Gilmore M. (2000). Relationships between enterococcal virulence and antimicrobial resistance. Clin. Microbiol. Rev..

[25] Adegoke A.A., Okoh A.I. (2014). Species diversity and antibiotic resistance properties of Staphylococcus of farm animal origin in Nkonkobe Municipality, South Africa. Folia Microbiol..

[26] Depardieu F., Perichon B., Courvalin P. (2004). Detection of the van alphabet and identification of enterococci and staphylococci at the species level by multiplex PCR. J. Clin. Microbiol..

[27] World Health Organization (2017). WHO Publishes List of Bacteria for Which New Antibiotics Are Urgently Needed. https://www.who.int/news/item/27-02-2017-who-publishes-list-of-bacteria-for-which-new-antibiotics-are-urgently-needed.

[28] Chuang O.N., Schlievert P.M., Wells C.L., Manias D.A., Tripp T.J. (2009). Multiple functional domains of *Enterococcus faecalis* aggregation substance Asc10 contribute to endocarditis virulence. Infect. Immun..

[29] Lee M.G., Kang M.J., Kim S., Jeong H., Kang D.K., Paik H.D., Park Y.S. (2024). Safety Assessment of *Levilactobacillus brevis* KU15006: A Comprehensive Analysis of its Phenotypic and Genotypic Properties. Prob. Antimicrob. Prot..

[30] Biswas P.P., Dey S., Sen A., Adhikan L. (2016). Molecular characterization of virulence genes in vancomycin-resistant and vancomycin-sensitive enterococci. J. Glob. Infect. Dis..

[31] Kim S.J., Shin S.Y., Kang S.J., Kim T.H. (2016). Biofilm formation and virulence factors in clinical *Enterococcus faecalis* isolates from patients with urinary tract infections in Korea. J. Med. Microbiol..

[32] Strateva T., Atanasova D., Savov E., Petrova G., Mitov I. (2016). Incidence of virulence determinants in clinical *Enterococcus faecalis* and *Enterococcus faecium* isolates collected in Bulgaria. Braz. J. Infect. Dis..

[33] Zhu Y.G., Johnson T.A., Su J.Q., Qiao M., Guo G.X., Stedtfeld R.D., Hashsham S.A., Tiedje J.M. (2013). Diverse and abundant antibiotic resistance genes in Chinese swine farms. Proc. Natl. Acad. Sci. USA.

[34] Bengtsson-Palme J., Kristiansson E., Larsson D.G.J. (2018). Environmental factors influencing the development and spread of antibiotic resistance. FEMS Microbiol. Rev..

[35] Larsson D.G.J. (2014). Pollution from drug manufacturing: Review and perspectives. Philos. Trans. R. Soc. Lond. B Biol. Sci..

[36] Heuer H., Schmitt H., Smalla K. (2011). Antibiotic resistance gene spread due to manure application on agricultural fields. Curr. Opin. Microbiol..

[37] Gaze W.H., Krone S.M., Larsson D.G., Li X.Z., Robinson J.A., Simonet P., Tiedje J.M. (2011). Influence of humans on evolution and mobilization of environmental antibiotic resistome. Emerg. Infect. Dis..

[38] Finley R.L., Collignon P., Larsson D.G., McEwen S.A., Li X.Z. (2013). The scourge of antibiotic resistance: The important role of the environment. Clin. Infect. Dis..

[39] Chee-Sanford J.C., Mackie R.I., Koike S., Krapac I.G., Lin Y.F., Yannarell A.C., Fate G.D. (2009). Fate and transport of antibiotic residues and antibiotic resistance genes following land application of manure waste. J. Environ. Qual..

[40] Muziasari W.I., Pitkänen L.K., Sorum H., Stedtfeld R.D., Tiedje J.M., Virta M. (2014). The resistome of farmed fish feces contributes to the enrichment of antibiotic resistance genes in sediments below marine fish farms. Front. Microbiol..

[41] Iweriebor B.C., Obi L.C., Okoh A.I. (2016). Macrolide, glycopeptide resistance and virulence genes in Enterococcus species isolates from dairy cattle. J. Med. Microbiol..

[42] Pignata C., Fea E., Rovere R., Degan R., Lorenzi E., de Ceglia M., Schilirò T., Gilli G. (2012). Chlorination in a wastewater treatment plant: Acute toxicity effects of the effluent and of the recipient water body. Environ. Monit. Assess..

[43] Osman K., Alvarez-Ordóñez A., Ruiz L., Badr J., ElHofy F., Al-Maary K.S., Moussa I.M., Hessain A.M., Orabi A., Saad A. (2017). Antimicrobial resistance and virulence characterization of Staphylococcus aureus and coagulase-negative staphylococci from imported beef meat. Ann. Clin. Microbiol. Antimicrob..

[44] Kotzamanidis C., Zdragas A., Kourelis A., Moraitou E., Papa A., Yiantzi V., Pantelidou C., Yiangou M. (2009). Characterization of vanA-type *Enterococcus faecium* isolates from urban and hospital wastewater and pigs. J. Appl. Microbiol..

[45] Li S., Zhou Y., He F., Raheem A., Yang H., Pan Y., Pan Z. (2023). Highly efficient capture of antibiotic resistance genes in wastewater using novel biochar-based hybrid adsorbents. Sci. Total Environ..

[46] Johnson A.P., Warner M., Hallas G., Livermore D.M. (2000). Susceptibility to quinupristin/dalfopristin and other antibiotics of van-comycin-resistant enterococci from the UK, 1997 to mid-1999. J. Antimicrob. Chemother..

[47] Martin J.F., Alvarez-Alvarez R., Liras P. (2022). Penicillin-binding proteins, β-lactamases, and β-lactamase inhibitors in β-lactam-producing actinobacteria: Self-resistance mechanisms. Int. J. Mol. Sci..

[48] Figueira V., Vaz-Moreira I., Silva M., Manaia C.M. (2011). Diversity and antibiotic resistance of *Aeromonas* spp. in drinking and wastewater treatment plants. Water Res..

[49] Said L.B., Klibi N., Lozano C., Dziri R., Slama K.B., Boudabous A., Torres C. (2015). Diversity of enterococcal species and characterization of high-level aminoglycoside resistant enterococci of samples of wastewater and surface water in Tunisia. Sci. Total Environ..

[50] Hashem Y.A., Abdelrahman K.A., Aziz R.K. (2021). Phenotype–genotype correlations and distribution of key virulence factors in *Enterococcus faecalis* isolated from patients with urinary tract infections. Infect. Drug Res..

[51] Kajihara T., Nakamura S., Iwanaga N., Oshima K., Takazono T., Miyazaki T., Izumikawa K., Yanagihara K., Kohno N., Kohno S. (2015). Clinical characteristics and risk factors of enterococcal infections in Nagasaki, Japan: A retrospective study. BMC Infect. Dis..

[52] Revathy S., Sridharan K.S., Elumalai A.S., Umasekar U. (2009). Phenotypic detection of high-level aminoglycoside resistance (HLAR) in Enterococcus species in a tertiary care centre. J. Clin. Diagn. Res..

[53] La-Rosa S.L., Montealegre M.C., Singh K.V., Murray B.E. (2016). *Enterococcus faecalis* Ebp pili are important for cell-cell aggregation and intraspecies gene transfer. Microbiology.

[54] Barbosa J., Gibbs P.A., Teixeira P. (2010). Virulence factors among enterococci isolated from traditional fermented meat products produced in the North of Portugal. Food Control.

[55] Rahman M.M., Hasan M., Ahmed A. (2021). Potential detrimental role of soluble ACE2 in severe COVID-19 comorbid patients. Rev. Med. Virol..

[56] Maheshwari M., Ahmad I., Althubiani A.S. (2016). Isolation and molecular characterization of multidrug-resistant *Enterococcus faecalis* from clinical samples. Trop. J. Pharm. Res..

[57] Chajęcka-Wierzchowska W., Zadernowska A., Łaniewska-Trokenheim Ł. (2017). Virulence factors of *Enterococcus* spp. presented in food. LWT.

[58] Gonzalez B., Pham P., Top J., Willems R.J.L., van Schaik W., van Passel M.W.J., Smidt H. (2017). Characterization of Enterococcus isolates colonizing the intestinal tract of intensive care unit patients receiving selective digestive decontamination. Front. Microbiol..

[59] Adegoke A.A., Faleye A.C., Stenstrom T.A. (2018). Residual antibiotics, antibiotic-resistant superbugs, and antibiotic resistance genes in surface water catchments: Public health impact. Phys. Chem. Earth.

[60] Vincent S., Minkler P., Bincziewski B., Etter L., Shlaes D.M. (1992). Vancomycin resistance in *Enterococcus gallinarum*. Antimicrob. Agents Chemother..

[61] Hao L., Wang H. (2024). Successful treatment of *Enterococcus gallinarum* infection in a neonate with vancomycin: A case report. BMC Pediatr..

[62] Tharvornvee W., Pruksakorn C., Lekcharoensuk P. (2016). Inducible vancomycin resistance is common in porcine *Enterococcus gallinarum* and *E. casseliflavus* isolates. Thai J. Vet. Med..

[63] García-Solache M., Rice L.B. (2019). The *Enterococcus*: A model of adaptability to its environment. Clin. Microbiol. Rev..

[64] Lioy P.J. (2010). Exposure science: A view of the past and milestones for the future. Environ. Health Perspect..

[65] Keraita B., Amoah P., Drechsel P., Scott C.A., Raschid-Sally L. (2011). Wastewater use in urban and peri-urban vegetable farming. Wastewater Irrigation and Health.

[66] Bonetta S., Pignata C., Gasparro E., Richiardi L., Bonetta S., Carraro E. (2022). Impact of wastewater treatment plants on microbiological contamination for evaluating the risks of wastewater reuse. Environ. Sci. Eur..

[67] Torres C., Alonso C.A., Ruiz-Ripa L., León-Sampedro R., Del Campo R., Coque T.M. (2018). Antimicrobial resistance in Enterococcus spp. of animal origin. Antimicrobial Resistance in Bacteria from Livestock and Companion Animals.

[68] European Centre for Disease Prevention and Control (ECDC) (2011). European Centre for Disease Prevention and Control Publishes Annual Epidemiological Report 2011. Eurosurveillance.

[69] Ammerlaan H.S., Harbarth S., Buiting A.G., Crook D.W., Fitzpatrick F., Hanberger H., Herwaldt L.A., Van Keulen P.H., Kluytmans J.A., Kola A. (2013). Secular trends in nosocomial bloodstream infections: Antibiotic-resistant bacteria increase the total burden of infection. Clin. Infect. Dis..

[70] De Kraker M.E., Jarlier V., Monen J.C., Heuer O.E., van de Sande N., Grundmann H. (2013). The changing epidemiology of bacteraemias in Europe: Trends from the European Antimicrobial Resistance Surveillance System. Clin. Microbiol. Infect..

[71] Didem K.A.R.T., Kuştimur A.S. (2019). Investigation of gelatinase gene expression and growth of *Enterococcus faecalis* clinical isolates in biofilm models. Turk. J. Pharm. Sci..

[72] Holá V., Ruzicka F., Horka M. (2010). Microbial diversity in biofilm infections of the urinary tract with the use of sonication techniques. FEMS Immunol. Med. Microbiol..

[73] Dworniczek E., Piwowarczyk J., Bania J., Kowalska-Krochmal B., Wałecka E., Seniuk A., Dolna I., Gościniak G. (2012). Enterococcus in wound infections: Virulence and antimicrobial resistance. Acta Microbiol. Immunol. Hung..

[74] Sava I., Heikens E., Toma I., Kropec A., Willems R., Hübner J. Enterococcal surface protein is a virulence factor in bacteremia but is not a target of opsonic antibodies in E. faecium infection. Proceedings of the American Society for Microbiology 109th General Meeting.

