# Supplementary material for: Virulence and Antibiotic Resistance Genes in Enterococcus from Wastewater for Reuse and Their Health Impact
Source: Microorganisms. 2025 Apr 30;13(5):1045. doi: 10.3390/microorganisms13051045 (PMC12114264; doi:10.3390/microorganisms13051045)
Supplement: Supplementary file 1 [file microorganisms-13-01045-s001.zip › microorganisms-3558131-supplementary.pdf]

Supplementary Table S1 List of primers for detection of resistance genes

| Gene | Primer sequence (5'-3') | Product size (bp) |
|---|---|---|
| *vanA* | F-GCGCGGTCCACTTGTAGATA | 314 |
| | R-TGAGCAACCCCCAAACAGTA | |
| *vanB* | F-AGACATTCCGGTCGAGGAAC | 220 |
| | R-GCTGTCAATTAGTGCGGGAA | |
| *vanC1* | F-ATCCAAGCTATTGACCCGCT | 402 |
| | R-TGTGGCAGGATCGTTTTCAT | |
| *vanC2/3* | F-CTAGCGCAATCGAAGCACTC | 582 |
| | R-GTAGGAGCACTGCGGAACAA | |
| *gyrA* | F-AAATCTGCCCGTGTCGTTGGT | 343 |
| | R-GCCATACCTACGGCGATACC | |
| *parC* | F-AAAAATCAGCGCGTACAGTG | 327 |
| | R-CGAGAGTTTGGCTTCGGTAT | |
| *tetA* | F-GCTACATCCTGCTTGCCTTC | 210 |
| | R-CATAGATCGCCGTGAAGAGG | |
| *tetB* | F-TTGGTTAGGGGCAAGTTTTG | 659 |
| | R-GTAATGGGCCAATAACACCG | |
| *tetC* | F-CTTGAGAGCCTTCAACCCAG | 418 |
| | R-ATGGTCGTCATCTACCTGCC | |
| *tetD* | F-AAACCATTACGGCATTCTGC | 787 |
| | R-GACCGGATACACCATCCATC | |
| *tetE* | F-AAACCACATCCTCCATACGC | 278 |
| | R-AAATAGGCCACAACCGTCAG | |
| *tetG* | F-GCTCGGTGGTATCTCTGCTC | 468 |
| | R-AGCAACAGAATCGGGAACAC | |
| *tetK* | F-TCGATAGGAACAGCAGTA | 169 |
| | R-CAGCAGATCCTACTCCTT | |
| *tetL* | F-TCGTTAGCGTGCTGTCATTC | 267 |
| | R-GTATCCCACCAATGTAGCCG | |
| *tetM* | F-GTATCCCACCAATGTAGCCG | 406 |
| | R-CGGTAAAGTTCGTCACACAC | |
| *tetO* | F-AACTTAGGCATTCTGGCTCAC | 515 |
| | R-TCCCACTGTTCCATATCGTCA | |
| *tetS* | F-CATAGACAAGCCGTTGACC | 667 |
| | R-ATGTTTTTGGAACGCCAGAG | |
| *tetA(P)* | F-CTTGGATTGCGGAAGAAGAG | 676 |
| | R-ATATGCCCATTTAACCACGC | |
| *tetQ* | F-TTATACTTCCTCCGGCATCG | 904 |

| | | |
|---|---|---|
| | R-ATCGGTTCGAGAATGTCCAC | |
| *tetX* | F-CAATAATTGGTGGTGGACCC | 468 |
| | R-TTCTTACCTTGGACATCCCG | |
| *emeA* | F-GTGACAGCCTTTGTGGCAGAT | 687 |
| | R-TAGTCCGTTGATGGTTCCTTG | |

In this study, the cocktail mixture consisted of 12.5 µL of PCR master mix (DreamTag MM Thermo Fisher), 20 µM for the primers, water, and 5 µL of template DNA, resulting in a total reaction volume of 25 µL. The amplification conditions involved an initial enzyme activation/denaturation at 94ºC for 5 minutes, followed by 35 cycles, with each cycle comprising 45 seconds at 94ºC, 60 s at 54ºC, and 90 s at 72ºC. A final elongation step was conducted for 15 minutes at 72ºC. The resulting amplicons were separated through electrophoresis in a 2% agarose gel at 110 V for 45 min, and the interpretation of results was based on the band sizes using a 100 bp ladder. with primer concentrations of 1.25 µM, 1.0 µM, 0.5 µM, 1.25 µM, and 0.5 µM, respectively. Group IV included primers for *tetA*(P) (1.25 µM), *tet*(Q) (1.25 µM) and *tet*(X) (1.25 µM) each). This was done in a reaction volume of 25 µL consisting of 12.5 µL master mix (OneTag Quick Load Master mix with standard buffer, New England BioLabs), and 5 µL of DNA template. Cycling conditions was as follows;

An initial denaturation at 94°C for 5 minutes was succeeded by 35 cycles, each consisting of 1 minute at 94°C, 1 minute at 55°C, and 1.5 minutes at 72°C.

Supplementary Table S2: Primers for the detection of virulence genes

| **Gene** | **Primer sequence (5'-3')** | **Product size (bp)** |
|---|---|---|
| *ace* | F- AAAGTAGAATTAGATCCACAC | 320 |
| | R- TCTATCACATTCGGTTGCG | |
| *asa1* | F-GCACGCTATTACGAACTATGA | 375 |
| | R-TAAGAAAGAACATCACCACGA | |
| *clyA* | F- ACTCGGGGATTGATAGGC | 688 |
| | R- GCTGCTAAAGCTGCGCTT | |

| | | |
|---|---|---|
| *efaA* | F- CGTGAGAAAGAAATGGAGGA<br>R- CTACTAACACGTCACGAATG | 499 |
| *esp* | F-AGATTTCATCTTTGATTCTTGG<br>R-AATTGATTCTTTAGCATCTGG | 510 |
| *hyl* | F- ACAGAAGAGCTGCAGGAAATG<br>R- GACTGACGTCCAAGTTTCCAA | 276 |
| *gelE* | F-TATGACAATGCTTTTTGGGAT<br>R-AGATGCACCCGAAATAATATA | 213 |

The PCR reaction volume, set at 20 µL, comprised 10 µL of OneTag rapid Load Master Mix (New England Bio Labs), 1 µL each of primers, and 5 µL of DNA template. For *gyrA*, the protocol included an initial template denaturation at 95°C for 1 minute, followed by 36 cycles of denaturation at 95°C for 30 seconds, annealing at 53°C for 30 seconds, and extension at 72°C for 2 minutes. A final elongation step was performed at 72°C for 10 minutes. Regarding *parC*, the conditions consisted of an initial template denaturation at 95°C for 2 minutes, with the next being 36 cycles of denaturation at 95°C for 60 s, annealing at 60°C for 60 s, and extension at 72°C for 120 s, with a final extension at 72°C for 10 minutes.

In a 20 µL reaction mixture, 10 µL of Quick Load Master Mix, 0.5 µL each of forward and reverse primers, 4 µL of DNA-free water, and 5 µL of DNA template were combined. The cycling conditions included an enzyme activation and initial denaturation at 94°C for 5 minutes, with the next being 30 cycles of denaturation at 94°C for 45 seconds, annealing at 57°C for 60 seconds, and extension at 72°C for 90 seconds, with a final elongation at 72°C for 10 minutes [14]. Electrophoresis of 5 µL of the PCR product on a 2% agarose gel with 1x TAE buffer was performed, dyed with Gel Red, and visualized using a UV transilluminator (BIORAD). The 100-bp ladder was utilized
